# New Treatment Paradigms Defined for Chronic Lymphocytic Leukemia

**DOI:** 10.6004/jadpro.2017.8.3.10

**Published:** 2017-04-01

**Authors:** Mollie Moran, Jeffrey Jones

**Affiliations:** The James Cancer Hospital at The Ohio State University, Columbus, Ohio

## Abstract

Within the past 3 years, the FDA has approved five new treatment options for patients with CLL. While these advances improve patient outcomes and the quality of care, there is a need for advanced practitioners to stay up to date and informed in order to translate these advances into the clinical setting.

Chronic lymphocytic leukemia (CLL) is the most common leukemia in the Western hemisphere, with 15,000 diagnoses each year and 4,400 deaths annually. Chronic lymphocytic leukemia is also the most prevalent leukemia because of its long survival time, with 60% of patients with CLL still alive 5 years after diagnosis and 35% alive at 10 years. Long-term survival has been possible because of advances in treatment, both in the front-line and relapsed settings. At JADPRO Live 2016, these treatments, along with predictors of outcome, were discussed by Mollie Moran, CNP, and Jeffrey Jones, MD, MPH, both of The James Cancer Hospital at The Ohio State University, Columbus, Ohio.

## CLINICAL STAGING AND PROGNOSIS

Clinical staging is an important factor in CLL (Table). Median survival is > 10 years with low-risk disease (Rai stage 0) but only 9 months to 4 years with high-risk disease (stage III and IV). By Binet group, which is more commonly used in Europe, survival is about 12 years in patients with group A clinical features (< 3 areas of lymphadenopathy and no anemia or thrombocytopenia) compared with only 2 to 4 years in patients with group C clinical features (anemia or thrombocytopenia).

**Table:**
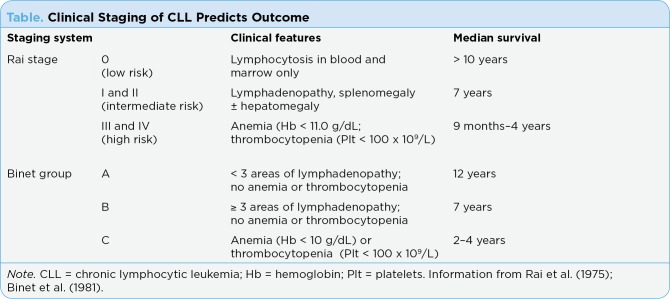
Clinical Staging of CLL Predicts Outcomez

Prognosis is also influenced by cellular, genetic, and nondisease-related factors, such as serum markers, lymphocyte doubling time, chromosomal aberrations (del[17p], del[11q]), genetic mutations (*IgVH* mutation status, *TP53*), age, gender, and health status.

In a study by Hamblin et al. involving 84 patients with CLL, median survival for patients with unmutated *IgVH* genes was 117 months versus 293 months for those with mutated *IgVH* (Hamblin, Davis, Gardiner, Oscier, & Stevenson, 1999). Survival is also poor in patients with del(17p), who tend not to respond to standard therapies, whereas patients with 13q deletion as their sole abnormality tend to have a good prognosis (Döhner et al., 2000).

## FIRST-LINE THERAPY

Indications for therapy in CLL include the extent and severity of disease manifestations. They include CLL-related symptoms such as night sweats, severe fatigue, and fever; progressive lymphadenopathy or progressive splenomegaly; a lymphocyte doubling time < 6 months; threatened end-organ function; bone marrow failure as indicated by progressive anemia or progressive thrombocytopenia; and immune dysfunction.

"We don’t treat patients until they are symptomatic…. Lymphocytosis alone is not a reason to treat a patient," said Ms. Moran. "It’s not a predictor of how patients will do with treatment."

The phase III CLL8 trial established the triplet of fludarabine, cyclophosphamide, and rituximab (Rituxan, FCR) as a superior treatment to doublet therapy with fludarabine and cyclophosphamide (FC) in untreated CLL (Hallek et al., 2010). In the study, 817 patients with CLL were randomized to receive FC or FCR. Median progression-free survival (PFS) was 51.8 months in the FCR arm vs. 32.8 months in the FC arm (hazard ratio [HR] = 0.563; *p* < .001), and 3-year overall survival (OS) rates were 87% and 82.5%, respectively (HR = 0.664; *p* = .012). The triplet FCR significantly improved the rate of complete response in patients with del(11q), del(13q), trisomy 12, and unmutated *IgVH* (*p* < .001) but not in those with del(17p; *p* = 0.3).

The CLL8 trial also demonstrated that "if you have two or more comorbidities, in addition to CLL, that affects your prognosis poorly," said Ms. Moran. For older patients and patients with comorbidities, bendamustine plus rituximab (BR) has often been the treatment of choice.

The CLL10 study randomized 557 patients with untreated active CLL in good physical condition to receive FCR or BR (Eichhorst et al., 2016). With a median observation time of 27.9 months, median PFS was longer in the FCR arm than in the BR arm in patients < 65 years (not reached vs. 36.5 months, *p* < .01), but this improvement was lost in the patients ≥ 65 years, for whom median PFS was 45.6 months with FCR and was not reached with BR. The difference may be attributable to the favorable toxicity profile with BR. Grade ≥ 3 hematologic toxicity, neutropenia, and infections were all significantly higher with FCR. In patients ≥ 65 years, the rates of ≥ 3 toxicity were 47% with FCR and 26% with BR in CLL10 (*p* = .002).

**Newer Agents**

Two anti-CD20 monoclonal antibodies have been approved since the approval of rituximab: ofatumumab (Arzerra) and obinutuzumab (Gazyva). The phase III CLL11 trial was an open-label three-arm study in which patients were randomized to receive chlorambucil alone (n = 118), chlorambucil plus obinutuzumab (n = 238), or chlorambucil plus rituximab (n = 233; Van Goede et al., 2014). Both combination arms produced better response rates, including complete responses, than chlorambucil alone. In a comparison of the combination arms, obinutuzumab/chlorambucil improved PFS more, but OS was similar in the two arms (Van Goede et al., 2015).

Since B-cell receptor signaling plays a major role in the development of CLL, interruption of this pathway with B-cell receptor pathway inhibitors represents a major advance in CLL treatment. In the open-label RESONATE-2 study, 269 treatment-naive patients ≥ 65 years old with CLL or small lymphocytic lymphoma were randomized to receive ibrutinib (Imbruvica) or chlorambucil (Burger et al., 2015).

At a median follow-up of 18.4 months, the PFS rate for patients assigned to ibrutinib was not reached, compared with 18.9 months for patients assigned to chlorambucil, corresponding to an 84% reduction in the risk of disease progression or death with ibrutinib (HR = 0.16, *p* < .0001). In addition, ibrutinib reduced the risk of death by 84% compared with chlorambucil (HR = 0.16, *p* = .001). The study served as the basis for approval of ibrutinib in the treatment of CLL.

Ibrutinib, however, was not without frequent but general mild side effects, especially diarrhea (42% vs. 17%), but also cough, peripheral edema, arthralgia, and dry eyes. "There’s also an issue of rash with ibrutinib, and it can be anything from just a red rash that doesn’t bother patients to big, purple nodules like erythema nodosum, which need to be treated with either dose reduction, a break from the drug, or a steroid," said Ms. Moran.

**NCCN Treatment Recommendations**

**Figure 1:**
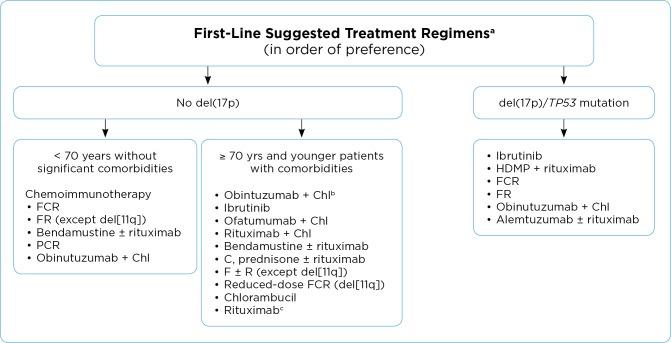
NCCN first-line therapy recommendations in CLL. Adapted from National Comprehensive Cancer Network. ^a^All recommendations category 2A unless otherwise stated; ^b^Category 1 recommendation; ^c^Category 3 recommendation. CLL = chronic lymphocytic leukemia; F = fludarabine; C = cyclophosphamide; R = rituximab; P = pentostatin; Chl = chlorambucil; HDMP = high-dose methylprednisolone.

As first-line treatment for CLL in patients without del(17p), the National Comprehensive Cancer Network (NCCN) suggests chemoimmunotherapy for patients < 70 years without significant comorbidities; it offers several choices for older patients and younger patients with comorbidities, including (in order of preference) obinutuzumab/chlorambucil, single-agent ibrutinib, ofatumumab/chloramabucil, rituximab/chlorambucil, and bendamustine with or without rituximab (Figure 1). For patients with del(17p) or TP53 mutation, NCCN recommends (in order of preference) ibrutinib, high-dose methylprednisolone/rituximab, FCR, fludarabine/rituximab, obinutuzumab/chlorambucil, and alemtuzumab (Campath) with or without rituximab.

## THERAPY FOR RELAPSED OR REFRACTORY DISEASE

Historically, patients with fludarabine-refractory CLL who are either refractory to alemtuzumab (double-refractory) or ineligible for alemtuzumab due to bulky lymphadenopathy had poor responses to subsequent lines of therapy. The overall response rates (ORR) in these two patient populations were 20% and 26%, respectively, and the median OS was 8 months and 14 months, respectively (Tam et al., 2007).

"Many of them had problems with persistent cytopenias and experienced a significant increase in the risk for infection, which was often fatal," Dr. Jones said.

In 2013, PFS rates with ibrutinib for the treatment of relapsed/refractory CLL were found to be similar across genetic risk groups (Byrd et al., 2013). "If you looked even at the patients with del(17p) CLL, their outcome with ibrutinib was twice as good as anything that had been reported for del(17p) patients, even in the front line," he noted. "For that reason, ibrutinib was approved not only for relapsed CLL on the basis of this study, but also for del(17p) CLL in the front line."

In an older study, ofatumumab was approved for the treatment of double-refractory CLL on the basis of an ORR of 50% and a median PFS of 6 months. Ibrutinib proved superior to ofatumumab in the RESONATE head-to-head comparison, not only in terms of PFS (HR = 0.215, *p* < .0001) but also OS (HR = 0.434, p = .0049); on this basis, ibrutinib received unrestricted approval for the treatment of relapsed or refractory CLL.

As ibrutinib treatment continues, patients tend to recover much of their bone marrow function, Dr. Jones added.

In RESONATE, atrial fibrillation of any grade was observed more frequently in patients receiving ibrutinib compared with ofatumumab (10 vs. 1 patient; Byrd et al., 2014); however, only one patient discontinued ibrutinib as a result. Bleeding-related adverse events of any grade (most commonly petechiae and bruising) were more common with ibrutinib than with ofatumumab (44% vs. 12%), although most were grade 1. Rates of severe/major bleeding events, however, were similar between ofatumumab and ibrutinib, and only one patient discontinued ibrutinib due to bleeding. Notably, some 37% of patients in the ibrutinib arm and 28% of patients in the ofatumumab arm received either concomitant antiplatelet agents (excluding nonsteroidal anti-inflammatory drugs) or anticoagulants.

Other B-cell receptor signaling antagonists have also been approved to treat relapsed CLL. Idelalisib (Zydelig) is an inhibitor of phosphatidylinositol 3 kinase p110ä. When given with rituximab, idelalisib improved PFS and OS compared with rituximab alone in patients with relapsed CLL (Furman et al., 2014). "Like ibrutinib, that benefit does not come without toxicity, and there are important toxicities with idelalisib as there are with ibrutinib," said Dr. Jones. "They’re not the typical toxicities that we experience with chemotherapy. It’s things like diarrhea…transaminitis, and, in some cases, problems with blood cell counts."

An alert for idelalisib was issued by the US Food and Drug Administration in March 2016. Because of an increased risk of infectious adverse events, including death, with idelalisib, new safety recommendations include institution of prophylaxis for *Pneumocystis carinii* pneumonia and cytomegalovirus infection and careful monitoring for neutropenia, with dose interruption if needed. Idelalisib is still approved in combination with rituximab for the treatment of relapsed CLL.

The most recently approved targeted therapy in CLL is venetoclax (Venclexta). Unlike B-cell receptor signaling drugs, which disconnect the accelerator from the engine of the CLL cell, venetoclax restores the "brakes" on cell proliferation and oncogenesis, Dr. Jones explained. Single-agent venetoclax in relapsed CLL, including high-risk patients, was associated with an 80% ORR, including some complete responses, in a phase I study (Roberts et al., 2016), which Dr. Jones noted "is decidedly uncommon with single-agent kinase inhibitors."

In a phase II study of patients with relapsed or refractory CLL with del(17p), the ORR to venetoclax monotherapy was 85%, and responses were durable (Stilgenbauer et al., 2015). Twelve-month estimates for PFS and OS were 72.0% and 86.7%, respectively.

Hyperacute tumor lysis leading to rapid changes in electrolytes and renal failure is a potential adverse effect of venetoclax. For this reason, careful titration of venetoclax, starting at 20 mg/day during the first week, is necessary, according to Dr. Jones. Venetoclax also poses a risk for neutropenia, which is responsive to granulocyte colony-stimulating factors.

Other newer agents include acalabrutinib, a second-generation Bruton’s tyrosine kinase (BTK) inhibitor that is more specific for the BTK enzyme than is ibrutinib and which may lead to fewer off-target adverse effects. While head-to-head comparisons are underway, a phase I/II study of acalabrutinib in relapsed CLL suggested that toxicities may be less than with ibrutinib while efficacy appears similar (Byrd et al., 2016), he said.

**Figure 2:**
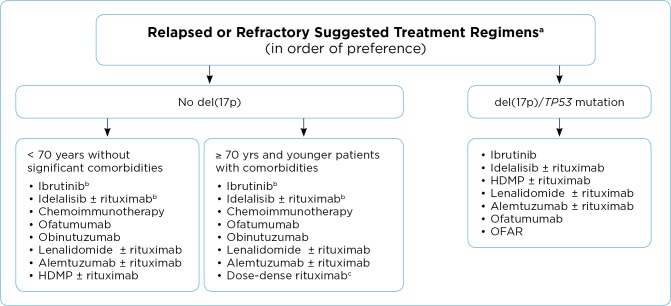
NCCN relapsed or refractory recommendations in CLL. Adapted from National Comprehensive Cancer Network. CLL = chronic lymphocytic leukemia; HDMP = high-dose methylprednisolone; OFAR = oxaliplatin, fludarabine, cytarabine, rituximab. ^a^All recommendations category 2A unless otherwise stated; ^b^Category 1 recommendation; ^c^Category 2B recommendation.

The NCCN therapy recommendations for relapsed or refractory CLL start with ibrutinib whether or not del(17p) is present (Figure 2). The second choice in all circumstances is idelalisib/rituximab. In those without del(17p), later choices (in order of preference) are chemoimmunotherapy, ofatumumab, obinutuzumab, lenalidomide (Revlimid)/rituximab, and alemtuzumab/rituximab.

## MANAGEMENT OF ADVERSE EVENTS

Patients with CLL often have comorbidities that require anticoagulant and/or antiplatelet agents, which can increase the risk of bleeding. Bleeding adverse events can lead to discontinuation of ibrutinib, most often when patients are taking one or more concomitant anticoagulant and/or antiplatelet agents. Therefore, withholding ibrutinib for 3 to 7 days before and after surgery should be considered, advised Dr. Jones.

As mentioned, atrial fibrillation can occur with ibrutinib, particularly among patients with cardiac risk factors. An electrocardiogram should be performed on any patient who develops symptoms of arrhythmia, such as palpitations or lightheadedness, or has new-onset dyspnea. Reducing the dose of ibrutinib if atrial fibrillation persists should be considered.

Diarrhea occurs frequently among patients on ibrutinib but is not usually treatment-limiting. Diarrhea can be managed symptomatically with over-the-counter antidiarrheals if necessary, but the complaint generally improves or resolves as treatment continues. For unusually severe symptoms, practitioners should consider interrupting ibrutinib treatment and work-up for other causes of diarrhea.

Idelalisib-related toxicities include diarrhea or colitis, transaminitis, and pneumonitis. Mild or moderate diarrhea or colitis usually occurs within the first 8 weeks of treatment and responds to common antidiarrheal agents. Grade 3 or 4 diarrhea is usually a late-occurring event and responds poorly to antidiarrheal or empiric antimicrobial therapy, in which case idelalisib should be withheld and the patient evaluated by colonoscopy. Steroid treatments such as oral budesonide are sometimes required to treat the colitis, and while reintroducing the drug at reduced dose has been attempted, many affected patients will permanently discontinue idelalisib treatment.

In terms of idelalisib-related transaminitis, most cases occur early (within the first 12 weeks). Liver enzyme elevations 5 to 20 times the upper limit of normal can often be managed by briefly withholding the drug and then reintroducing it at a lower dosage (100 mg/day vs. 150 mg twice daily). All pulmonary symptoms should be evaluated for pneumonitis; if it is suspected, idelalisib should be interrupted until the cause is determined, Dr. Jones indicated.

Patients taking obinutuzumab or venetoclax should be monitored frequently for neutropenia. White blood cell growth factor support should be instituted throughout treatment for patients with severe neutropenia, with consideration for antiviral and antifungal prophylaxis, as well.

## References

[1] Binet J L, Auquier A, Dighiero G, Chastang C, Piguet H, Goasguen J, Vaugier G, Potron G, Colona P, Oberling F, Thomas M, Tchernia G, Jacquillat C, Boivin P, Lesty C, Duault M T, Monconduit M, Belabbes S, Gremy F (1981). A new prognostic classification of chronic lymphocytic leukemia derived from a multivariate survival analysis.. *Cancer*.

[2] Burger Jan A, Tedeschi Alessandra, Barr Paul M, Robak Tadeusz, Owen Carolyn, Ghia Paolo, Bairey Osnat, Hillmen Peter, Bartlett Nancy L, Li Jianyong, Simpson David, Grosicki Sebastian, Devereux Stephen, McCarthy Helen, Coutre Steven, Quach Hang, Gaidano Gianluca, Maslyak Zvenyslava, Stevens Don A, Janssens Ann, Offner Fritz, Mayer Jiří, O’Dwyer Michael, Hellmann Andrzej, Schuh Anna, Siddiqi Tanya, Polliack Aaron, Tam Constantine S, Suri Deepali, Cheng Mei, Clow Fong, Styles Lori, James Danelle F, Kipps Thomas J (2015). Ibrutinib as Initial Therapy for Patients with Chronic Lymphocytic Leukemia.. *The New England journal of medicine*.

[3] Byrd John C, Brown Jennifer R, O'Brien Susan, Barrientos Jacqueline C, Kay Neil E, Reddy Nishitha M, Coutre Steven, Tam Constantine S, Mulligan Stephen P, Jaeger Ulrich, Devereux Steve, Barr Paul M, Furman Richard R, Kipps Thomas J, Cymbalista Florence, Pocock Christopher, Thornton Patrick, Caligaris-Cappio Federico, Robak Tadeusz, Delgado Julio, Schuster Stephen J, Montillo Marco, Schuh Anna, de Vos Sven, Gill Devinder, Bloor Adrian, Dearden Claire, Moreno Carol, Jones Jeffrey J, Chu Alvina D, Fardis Maria, McGreivy Jesse, Clow Fong, James Danelle F, Hillmen Peter (2014). Ibrutinib versus ofatumumab in previously treated chronic lymphoid leukemia.. *The New England journal of medicine*.

[4] Byrd John C, Furman Richard R, Coutre Steven E, Flinn Ian W, Burger Jan A, Blum Kristie A, Grant Barbara, Sharman Jeff P, Coleman Morton, Wierda William G, Jones Jeffrey A, Zhao Weiqiang, Heerema Nyla A, Johnson Amy J, Sukbuntherng Juthamas, Chang Betty Y, Clow Fong, Hedrick Eric, Buggy Joseph J, James Danelle F, O'Brien Susan (2013). Targeting BTK with ibrutinib in relapsed chronic lymphocytic leukemia.. *The New England journal of medicine*.

[5] Byrd John C, Harrington Bonnie, O'Brien Susan, Jones Jeffrey A, Schuh Anna, Devereux Steve, Chaves Jorge, Wierda William G, Awan Farrukh T, Brown Jennifer R, Hillmen Peter, Stephens Deborah M, Ghia Paolo, Barrientos Jacqueline C, Pagel John M, Woyach Jennifer, Johnson Dave, Huang Jane, Wang Xiaolin, Kaptein Allard, Lannutti Brian J, Covey Todd, Fardis Maria, McGreivy Jesse, Hamdy Ahmed, Rothbaum Wayne, Izumi Raquel, Diacovo Thomas G, Johnson Amy J, Furman Richard R (2016). Acalabrutinib (ACP-196) in Relapsed Chronic Lymphocytic Leukemia.. *The New England journal of medicine*.

[6] Döhner H, Stilgenbauer S, Benner A, Leupolt E, Kröber A, Bullinger L, Döhner K, Bentz M, Lichter P (2000). Genomic aberrations and survival in chronic lymphocytic leukemia.. *The New England journal of medicine*.

[7] Eichhorst Barbara, Fink Anna-Maria, Bahlo Jasmin, Busch Raymonde, Kovacs Gabor, Maurer Christian, Lange Elisabeth, Köppler Hubert, Kiehl Michael, Sökler Martin, Schlag Rudolf, Vehling-Kaiser Ursula, Köchling Georg, Plöger Christoph, Gregor Michael, Plesner Torben, Trneny Marek, Fischer Kirsten, Döhner Harmut, Kneba Michael, Wendtner Clemens-Martin, Klapper Wolfram, Kreuzer Karl-Anton, Stilgenbauer Stephan, Böttcher Sebastian, Hallek Michael (2016). First-line chemoimmunotherapy with bendamustine and rituximab versus fludarabine, cyclophosphamide, and rituximab in patients with advanced chronic lymphocytic leukaemia (CLL10): an international, open-label, randomised, phase 3, non-inferiority trial.. *The Lancet. Oncology*.

[8] Furman Richard R, Sharman Jeff P, Coutre Steven E, Cheson Bruce D, Pagel John M, Hillmen Peter, Barrientos Jacqueline C, Zelenetz Andrew D, Kipps Thomas J, Flinn Ian, Ghia Paolo, Eradat Herbert, Ervin Thomas, Lamanna Nicole, Coiffier Bertrand, Pettitt Andrew R, Ma Shuo, Stilgenbauer Stephan, Cramer Paula, Aiello Maria, Johnson Dave M, Miller Langdon L, Li Daniel, Jahn Thomas M, Dansey Roger D, Hallek Michael, O'Brien Susan M (2014). Idelalisib and rituximab in relapsed chronic lymphocytic leukemia.. *The New England journal of medicine*.

[9] Hallek M, Fischer K, Fingerle-Rowson G, Fink A M, Busch R, Mayer J, Hensel M, Hopfinger G, Hess G, von Grünhagen U, Bergmann M, Catalano J, Zinzani P L, Caligaris-Cappio F, Seymour J F, Berrebi A, Jäger U, Cazin B, Trneny M, Westermann A, Wendtner C M, Eichhorst B F, Staib P, Bühler A, Winkler D, Zenz T, Böttcher S, Ritgen M, Mendila M, Kneba M, Döhner H, Stilgenbauer S (2010). Addition of rituximab to fludarabine and cyclophosphamide in patients with chronic lymphocytic leukaemia: a randomised, open-label, phase 3 trial.. *Lancet (London, England)*.

[10] Hamblin T J, Davis Z, Gardiner A, Oscier D G, Stevenson F K (1999). Unmutated Ig V(H) genes are associated with a more aggressive form of chronic lymphocytic leukemia.. *Blood*.

[11] National Comprehensive Cancer Network (NCCN) (2016). NCCN Clinical Practice Guidelines in Oncology: Chronic Lymphocytic Leukemia/Small Lymphocytic Leukemia. v2.2016. https://www.nccn.org/professionals/physician_gls/pdf/cll.pdf.

[12] Rai K R, Sawitsky A, Cronkite E P, Chanana A D, Levy R N, Pasternack B S (1975). Clinical staging of chronic lymphocytic leukemia.. *Blood*.

[13] Roberts Andrew W, Davids Matthew S, Pagel John M, Kahl Brad S, Puvvada Soham D, Gerecitano John F, Kipps Thomas J, Anderson Mary Ann, Brown Jennifer R, Gressick Lori, Wong Shekman, Dunbar Martin, Zhu Ming, Desai Monali B, Cerri Elisa, Heitner Enschede Sari, Humerickhouse Rod A, Wierda William G, Seymour John F (2016). Targeting BCL2 with Venetoclax in Relapsed Chronic Lymphocytic Leukemia.. *The New England journal of medicine*.

[14] Stilgenbauer S, Eichhorst B F, Schetelig J, Coutre S, Seymour J F, Munir T, Wierda W (2015). Venetoclax (ABT-199/GDC-0199) monotherapy induces deep remissions, including complete remission and undetectable MRD, in ultra-high risk relapsed/refractory chronic lymphocytic leukemia with 17p deletion: Results of the pivotal international phase 2 study [Abstract LBA-6]. *Blood (ASH Annual Meeting Abstracts)*.

[15] Tam Constantine S, O'Brien Susan, Lerner Susan, Khouri I, Ferrajoli A, Faderl S, Browning M, Tsimberidou Apostolia M, Kantarjian Hagop, Wierda William G (2007). The natural history of fludarabine-refractory chronic lymphocytic leukemia patients who fail alemtuzumab or have bulky lymphadenopathy.. *Leukemia ’ lymphoma*.

[16] Van Goede V, Fischer K, Bosch F, Follows G, Frederiksen H, Cuneo A, Hallek M (2015). Updated survival analysis from the CLL11 study: Obinutuzumab versus rituximab in chemoimmunotherapy-treated patients with chronic lymphocytic leukemia. *Blood*.

[17] Van Goede Valentin, Fischer Kirsten, Busch Raymonde, Engelke Anja, Eichhorst Barbara, Wendtner Clemens M, Chagorova Tatiana, de la Serna Javier, Dilhuydy Marie-Sarah, Illmer Thomas, Opat Stephen, Owen Carolyn J, Samoylova Olga, Kreuzer Karl-Anton, Stilgenbauer Stephan, Döhner Hartmut, Langerak Anton W, Ritgen Matthias, Kneba Michael, Asikanius Elina, Humphrey Kathryn, Wenger Michael, Hallek Michael (2014). Obinutuzumab plus chlorambucil in patients with CLL and coexisting conditions.. *The New England journal of medicine*.

